# Prevalence, correlates, and quality-of-life outcomes of major or persistent pain among women living with HIV in Metro Vancouver, Canada

**DOI:** 10.1186/s12954-023-00859-x

**Published:** 2024-01-13

**Authors:** Sophia Ly, Kate Shannon, Melissa Braschel, Haoxuan Zhou, Andrea Krüsi, Kathleen Deering

**Affiliations:** 1 Division of Social Medicine, Department of Medicine, Faculty of Medicine, University of British Columbia, 1190 Hornby St, Vancouver, BC V6Z 2K5 Canada; 2 Centre for Gender and Sexual Health Equity, Vancouver, Canada; 3 Faculty of Medicine, University of British Columbia, Vancouver, Canada

**Keywords:** Women living with HIV, Chronic pain, Substance use, Poverty, Mental health, Biopsychosocial model

## Abstract

While women living with HIV (WLWH) are twice as likely to report severe or undertreated chronic pain compared to men, little is known about pain among WLWH. Our goal was to characterize the correlates of pain as well as its impact on quality-of-life outcomes among women enrolled in the Sexual Health and HIV/AIDS Women’s Longitudinal Needs Assessment (SHAWNA), an open longitudinal study of WLWH accessing care in Metro Vancouver, Canada. We conducted logistic regression analyses to identify associations between self-reported major or persistent pain with sociostructural and psychosocial correlates and with quality-of-life outcomes. Data are presented as adjusted odds ratios (aORs) with 95% confidence intervals. Among 335 participants, 77.3% reported pain at ≥ 1 study visit, with 46.3% experiencing any undiagnosed pain and 53.1% managing pain with criminalized drugs. In multivariable analysis, age (aOR 1.04[1.03–1.06] per year increase), food and housing insecurity (aOR 1.54[1.08–2.19]), depression diagnosis (aOR 1.34[1.03–1.75]), suicidality (aOR 1.71[1.21–2.42]), and non-daily, non-injection opioid use (aOR 1.53[1.07–2.17]) were associated with higher odds of pain. Daily non-injection opioid use (aOR 0.46[0.22–0.96]) and health services access (aOR 0.63[0.44–0.91]) were associated with lower odds of pain. In separate multivariable confounder models, pain was associated with reduced odds of good self-rated health (aOR 0.64[0.48–0.84] and increased odds of health interference with social activities (aOR 2.21[1.63–2.99]) and general function (aOR 3.24[2.54–4.13]). In conclusion, most WLWH in our study reported major or persistent pain. Pain was commonly undiagnosed and associated with lower quality of life. We identified structural and psychosocial factors associated with pain in WLWH, emphasizing the need for low-barrier, trauma-informed, and harm reduction-based interventions.

## Introduction

Pain is a major concern for many people living with HIV (PLWH). Any major or persistent pain may be associated with emotional distress and functional impairment among PLWH [21, 67]. Much of the existing work on major or persistent pain in PLWH centres on chronic pain (i.e. pain that lasts more than 3 months [72]), which has a prevalence of 54–83% among PLWH in North America [59] compared to 21% in the general Canadian population [69]. Common etiologies include HIV-related peripheral neuropathy; central sensitization syndromes, potentially mediated by HIV-associated inflammation of both nervous and peripheral tissues; antiretroviral side effects; and chronic musculoskeletal disorders (e.g. osteoarthritis) [29, 40, 42]. Chronic pain in PLWH is associated with adverse outcomes along the HIV care continuum, including sub-optimal antiretroviral therapy (ART) adherence [42, 67], increased disability, and reduced quality of life [30, 60]. Its impacts among PLWH may increase as HIV continues to evolve worldwide from a terminal condition into a chronic illness requiring long-term symptom management [2].

Pain is a multifactorial experience that benefits from a multidisciplinary, biopsychosocial treatment model [35]. This is particularly relevant in the context of pain experienced by PLWH. A 2021 systematic review remarked on the low efficacy of analgesic medications in randomized control trials on HIV-related pain [61], with two studies reporting 50–65% symptom relief on analgesic therapy [47, 49]. A comprehensive understanding of the key psychosocial and sociostructural factors contributing to pain among PLWH may therefore facilitate the development of more effective interventions. Previously identified psychological correlates of pain in PLWH include anxiety, depression, post-traumatic stress, and substance use disorder [20, 30, 53, 67]. Sociostructural correlates of pain in PLWH are less well characterized despite evidence that social interactions modulate the experience of pain [34] and structural inequities among PLWH limit access to care [19].

Women living with HIV (WLWH) are twice as likely to report severe pain compared to men with HIV [30]. This disparity has been hypothesized to arise from a combination of biological factors, such as sex differences in pain modulation and pharmacological response [4], as well as sociostructural factors, such as increased gender-based violence, intersectional discrimination, and other barriers to care [25], with WLWH twice as likely to have their pain undertreated compared to men [7]. The potential significance of sociostructural drivers in pain among WLWH in Canada, where WLWH represent more than one-quarter of PLWH [12], is corroborated by findings that Canadian WLWH experience poorer quality of care [13] and greater HIV-associated reductions in life expectancy [27] than their male counterparts.

These factors highlight the importance of a gendered analysis to understanding women’s needs for pain treatment as well as the impacts of pain on women’s health and well-being. Despite the high prevalence and disease burden of pain among WLWH, few studies have examined the specific correlates or outcomes of pain within this population. A 2018 systematic review of psychosocial factors associated with persistent pain in HIV noted that only 5 of 46 studies recruited predominantly WLWH [67], of which 2 studies examined social correlates of pain and 4 examined functional outcomes. Furthermore, it is unclear whether these studies included transgender (trans) WLWH or non-binary persons, reflecting the frequent erasure of gender minority communities from health research despite the unique inequities affecting these populations [54]. We have also been unable to identify studies examining the relationship between interpersonal violence and pain in HIV although there is a documented association between violence and pain in the general population [78] and a high prevalence of violence among WLWH [11, 19].

To better characterize pain in WLWH, our objectives were to examine: 1) the prevalence and correlates of self-reported major or persistent pain, herein referred to as “pain”, and 2) the association between pain and quality of life among WLWH in Metro Vancouver, Canada.

## Methods

### Study design and sampling

Data for this study were drawn over five years (September 2014–August 2019) from the Sexual Health and HIV/AIDS Women’s Longitudinal Needs Assessment (SHAWNA), an ongoing community-based, longitudinal open enrolment cohort study. SHAWNA was launched in 2014 to investigate the sociostructural factors mediating access to care for cisgender (cis) and trans (inclusive of transgender, transsexual, other transfeminine identity) WLWH. The study was developed through extensive community consultation with WLWH, HIV care providers, and policy experts. SHAWNA represents a partnership of community and HIV organizations and is informed by two advisory boards: a Community Stakeholder Advisory Board, and a Positive Women’s Advisory Board, comprised of WLWH who meet every two to three months.

Eligibility criteria included: self-identifying as a cis or trans woman, being 14 years of age or older, having a HIV diagnosis as established by confirmatory testing, and living and/or accessing HIV/AIDS services in Metro Vancouver. Participants were recruited by self-referral; referrals from HIV care providers, peer navigators, and HIV/AIDS advocacy groups (e.g. Canadian Aboriginal AIDS Network); and clinical outreach by partner organizations such as Oak Tree Clinic, the primary referral centre for WLWH in British Columbia.

Participants provided informed consent and completed a questionnaire at baseline and every six months on a range of sociostructural (e.g. trauma, violence, stigma, income, housing security), health (e.g. symptoms, treatments, access to care), and sociodemographic (e.g. age, race, sexual identity) variables. Questionnaires were administered by trained community interviewers and followed by a visit with a sexual health research nurse who offered HIV viral load/CD4 count monitoring, testing for sexually transmitted infections and hepatitis C, and referrals to health and social services. Participants received $50 CAD for each visit as compensation for their time and expertise. All tests and referrals were voluntary and did not affect research study participation or compensation. Ethics approval for this study was granted by the Providence Health/University of British Columbia Research Ethics Board and BC Women’s Hospital.

### Study measures

#### Primary variable of interest

Participants reported whether they experienced pain over the last 6 months at each study visit (time updated) by responding to the following question, modified from the Brief Pain Inventory Short Form (BPI-SF) [48], “Throughout our lives, most of us have had pain from time to time. In the last 6 months, have you had any major or persistent pain (other than minor headaches, sprains, etc.)?”. The BPI-SF has been widely used to characterize pain severity and interference in people with HIV [30, 49, 60, 68]. Subsequently, they were asked, “Has this pain been diagnosed by a doctor?” and “In the last 6 months, have you taken medication for this pain? Was this prescribed medication, over the counter (OTC) or illicit drugs?”.

#### Explanatory variables and potential confounders

Potential explanatory variables (i.e. correlates) of pain were selected based on a literature review. *Sociodemographic factors* included a variable measuring sexual orientation drawn from the question, “In the last 6 months, which of the following describes your sexual orientation (check all that apply)” and defined as sexual minority at any study visit (lesbian, gay, bisexual, queer, asexual, and/or Two-Spirit) versus only heterosexual at all study visits, as well as a variable measuring gender identity drawn from the question, “In the last 6 months, which of the following best describe(s) your gender identity (check all that apply)” and defined as gender minority at any study visit (trans [transgender, transsexual, other transfeminine identity], non-binary [non-binary, genderqueer], and/or Two-Spirit) versus only cisgender at all visits. Two-Spirit is an identity among people Indigenous to Turtle Island who identify as having both a masculine and a feminine spirit, and may be used to describe any or all of sexual, gender, and/or spiritual identity depending on the individual and context [62]. Participants had the option to provide more than one response to questions on sexual orientation and gender identity. Based on evidence that minority stress processes affect all gender minority people relative to cis people [70] and all sexual minority people relative to heterosexual people [51], for the purposes of analyses, we combined participants with responses to sexual minority identities into one variable and gender minority identities into one variable.

Additional sociodemographic variables included and race (Indigenous [First Nations, Métis, or Inuit], other racialized persons [African/Caribbean/Black, Latin American, East/South/Southeast Asian, Middle Eastern, or other visible minority], White). The term Indigenous is used throughout while recognizing great diversity across and within languages, cultures, nations, and lands. While descriptive data were disaggregated, given the small sample size of Black participants, comparable to the BC population, Black women and otherwise racialized women were combined in modelling to understand experiences of racism for non-Indigenous racialized persons. Additional variables included age (measuring continuously in years) high school graduation at baseline; residence in the Vancouver Downtown Eastside, a highly marginalized community where high rates of poverty, unstable housing, substance use, and survival sex work have contributed to an estimated HIV prevalence of 30% [38]; homelessness (having no place to sleep for at least 1 night) (last 6 months), food insecurity (responding often true or sometimes true to any item on a modified Cornell-Radimer Hunger Scale [31] as previously described [3]) (last 6 months), housing insecurity (meeting the Canadian Observatory of Homelessness definition [22] of unsheltered or otherwise unstably housed as previously described [79]) (last 6 months). A composite food and/or housing insecurity variable (food and housing secure, food or housing insecure, or food and housing insecure) (last 6 months) was also assessed given previous evidence that separate versus concurrent food and housing insecurity may be associated with different sociostructural inequities among Canadian WLWH [39]. *Mental health factors* included feeling downhearted or blue (drawn from the Medical Outcomes Study SF-36 survey [77] and defined as a response of all the time, most of the time, or a good bit of the time versus some of the time, a little of the time, or none of the time) (last 4 weeks), depression (receiving diagnosis and/or treatment) (last 6 months), and suicidal ideation (contemplating and/or attempting suicide) (last 6 months). *Substance use factors* included non-injection opioid use (daily, less than daily [more than once a week, once a week, 1–3 times per month, less than once per month], none) (last 6 months), injection opioid use (daily, less than daily [more than once a week, once a week, 1–3 times per month, less than once per month], none) (last 6 months), cannabis use (daily, less than daily [more than once a week, once a week, 1–3 times per month, less than once per month], none) (last 6 months), and accidental overdose (last 6 months). Our analysis focused on opioids and cannabis versus other criminalized substances as both have analgesic effects and previous work has demonstrated that people who use criminalized drugs in British Columbia may turn to non-prescription opioids and cannabis for pain management [14, 36]. Further, people who use injection opioid in particular may face increased stigma from healthcare providers limiting access to pain care [75]. *General health factors* included ability to access health services when needed (always or usually versus sometimes, occasionally, or never) (last 6 months) and detectable HIV-1 viral load (any test ≥ 50 copies/ml) (last 6 months). *Interpersonal factor*s included sexual violence by any perpetrator (last 6 months) and physical violence by any perpetrator (last 6 months). All variables were time updated at each semiannual study visit, except for race and high school graduation.

### Quality-of-life outcomes

Time updated quality-of-life outcome variables were drawn from the Medical Outcomes Study SF-36 survey [77] and included good self-rated health over the last 6 months (assessed with the question, “In general, how would you rate your health?” and defined as a response of excellent, very good, or good versus fair or poor), interference of health with social activities over the last 4 weeks (assessed with the question, “How much of the time during the past 4 weeks has your physical or emotional health interfered with your social activities?” and defined as a response of all the time, most of the time, or a good bit of the time versus some of the time, a little of the time, or none of the time), and interference of health with general function over the last 4 weeks (defined as answering yes to either of the questions, “During the past 4 weeks, have you accomplished less than you would like as a result of your physical health?” or “During the past 4 weeks, have you accomplished less than you would like as a result of your emotional health?” versus no to both). The decision was made not to administer the entire SF-36 survey due to concerns raised in community consultation that the full validated scale had not been developed for marginalized people and that several items contained language likely to be perceived as discriminatory or exclusive by study participants.

### Statistical analysis

Statistical analysis was performed using SAS software (version 9.4; SAS Institute Inc., Cary, NC). Descriptive statistics (i.e. frequency and per cent or median and interquartile range [IQR]) were calculated for all variables at baseline and stratified by pain in the last 6 months. Differences were assessed using Wilcoxon rank-sum tests for continuous variables and Pearson's Chi-square tests (or Fisher’s exact tests where cell counts were small) for categorical variables (Table 1).

**Table 1:** Baseline characteristics among cohort of 335 women living with HIV in Metro Vancouver, Canada, stratified by whether major or persistent pain was experienced in the last 6 months

	Overall (n) (N = 335)	Pain		P value	Missing (n)
		Any major or persistent pain^b^ (N = 161)	No major or persistent pain^b^ (N = 174)		
*Sociodemographic factors*					
Age (median, IQR) (years)	45 (38–52)	45 (39–52)	44 (37–50)	0.261	0
Sexual minority identity	40.6% (136)	42.2% (68)	39.1% (68)	0.525	1
Gender minority identity	10.5% (35)	10.6% (17)	10.3% (18)	0.962	3
Race				0.488	0
Indigenous	55.5% (186)	52.2% (84)	58.6% (102)		
Otherwise racialized person	10.2% (34)	11.2% (18)	9.2% (16)		
White	34.3% (115)	36.6% (59)	32.2% (56)		
Graduated high school	50.8% (170)	51.6% (83)	50.0% (87)	0.776	0
Currently living in Downtown Eastside^a^	23.6% (79)	21.7% (35)	25.3% (44)	0.427	1
Food insecurity^b^	70.8% (237)	71.4% (115)	70.1% (122)	0.920	2
Housing insecurity^b^	66.9% (224)	68.9% (111)	64.9% (113)	0.437	0
Homeless^b^	18.2% (61)	17.4% (28)	19.0% (33)	0.709	0
Food and housing insecurity^b^				0.063	2
Food or housing insecure	37.0% (124)	42.2% (68)	32.2% (56)		
Food and housing insecure	50.2% (168)	49.1% (79)	51.2% (89)		
*Mental health and substance use factors*					
Non-injection opioid use^b^				0.010	2
Daily	4.5% (15)	1.9% (3)	6.9% (12)		
Less than daily	10.5% (35)	14.3% (23)	6.9% (12)		
None	84.5% (283)	82.6% (133)	86.2% (150)		
Injection opioid use^b^				0.533	1
Daily	15.2% (51)	17.4% (28)	13.2% (23)		
Less than daily	17.3% (58)	17.4% (28)	17.2% (30)		
None	67.2% (225)	64.6% (104)	69.5% (121)		
Cannabis use^b^				0.178	2
Daily	15.2% (51)	12.4% (20)	17.8% (31)		
Less than daily	16.7% (56)	19.9% (32)	13.8% (24)		
None	67.5% (226)	67.1% (108)	67.8% (118)		
Accidental overdose^b^	5.4% (18)	5.6% (9)	5.2% (9)	0.855	1
Diagnosed/treated for depression^b^	29.3% (98)	32.3% (52)	26.4% (46)	0.239	0
Suicidal ideation^b^	11.0% (37)	13.7% (22)	8.6% (15)	0.119	8
*General health factors*					
Access to health services when needed^b^	88.4% (296)	82.6% (133)	93.7% (163)	0.002	0
Detectable viral load^b^	30.5% (102)	36.0% (58)	25.3% (44)	0.025	56
*Interpersonal factors*					
Sexual violence^b^	4.8% (16)	6.2% (10)	3.5% (6)	0.241	24
Physical violence^b^	12.5% (42)	12.4% (20)	12.6% (22)	0.911	19
*Quality-of-life outcomes*					
Good self-rated health^b^	69.6% (223)	64.0% (103)	74.7% (130)	0.033	0
Health interference with social activities^c^	29.3% (93)	38.2% (58)	21.1% (35)	< 0.001	0
Health interference with general function^c^	65.4% (208)	82.2% (125)	50.0% (83)	< 0.0001	1

^a^A highly marginalized Vancouver community with high rates of poverty, unstable housing, substance use, survival sex work, and HIV infection

^b^In the last 6 months

^c^In the last 4 weeks, and restricted to 2019 February. The number of participants in overall, any major or persistent pain, and no major or persistent pain are 318,  152, and 166 respectively.

Bivariate and multivariable logistic regression with generalized estimating equations (GEE), which use an exchangeable correlation structure to account for repeated measurements among participants, were performed to identify associations between explanatory variables and pain as the outcome (Table 2). The GEE approach uses a complete case analysis to account for missing data, whereby observations with any missing data on a given variable are excluded from the multivariable analysis. An explanatory multivariable model was generated using a manual backward elimination process. Hypothesized explanatory variables with p < 0.10 in bivariate analysis were considered for inclusion in the full multivariable model and assessed for multicollinearity using the variance inflation factor (VIF). Due to concerns about multicollinearity, the individual food insecurity and housing insecurity variables were omitted from the multivariable analysis with only the composite food and housing insecurity variable retained as a potential covariate. The variable with the largest p value of Type-III analysis was removed and the quasi-likelihood under the independence model criterion (QIC) was noted as previously described [18, 57]. The final model represented the one with the lowest QIC value, indicating the best model fit.

**Table 2:** Bivariate and multivariable odds ratios between potential correlates and major or persistent pain in the last 6 months among cohort of 335 women living with HIV in Metro Vancouver, Canada

	OR [95%CI]	P value	aOR [95%CI]	P value
*Sociodemographic factors*				
Age (per year older)	1.04 [1.02–1.06]	< 0.001	1.04 [1.03–1.06]	< 0.001
Sexual minority identity	1.30 [0.95–1.78]	0.097		
Gender minority identity	0.82 [0.48–1.40]	0.467		
Race				
Indigenous	1.06 [0.77–1.47]	0.715		
Otherwise racialized person	1.06 [0.62–1.82]	0.825		
White	Reference			
Graduated high school	0.81 [0.60–1.10]	0.183		
Currently living in Downtown Eastside^a^	0.78 [0.58–1.05]	0.106		
Food insecurity^b^	1.15 [0.90–1.48]	0.259		
Housing insecurity^b^	1.41 [1.16–1.72]	< 0.001		
Homeless^b^	0.98 [0.71–1.35]	0.888		
Food and housing insecurity^b^				
Food and housing secure	Reference		Reference	
Food or housing insecure	1.36 [1.01–1.85]	0.046	1.24 [0.89–1.71]	0.201
Food and housing insecure	1.70 [1.22–2.35]	0.002	1.54 [1.08–2.19]	0.017
*Mental health and substance use factors*				
Non-injection opioid use^b^				
Daily	0.51 [0.26–0.98]	0.045	0.46 [0.22–0.96]	0.039
Less than daily	1.51 [1.10–2.07]	0.010	1.53 [1.07–2.17]	0.019
None	Reference		Reference	
Injection opioid use^b^				
Daily	1.15 [0.81–1.64]	0.443		
Less than daily	0.95 [0.69–1.30]	0.740		
None	Reference			
Cannabis use^b^				
Daily	1.14 [0.82–1.57]	0.437		
Less than daily	1.23 [0.91–1.65]	0.172		
None	Reference			
Accidental overdose^b^	1.18 [0.85–1.64]	0.311		
Diagnosed/treated for depression^b^	1.33 [1.06–1.67]	0.015	1.34 [1.03–1.75]	0.030
Suicidal ideation^b^	1.86 [1.36–2.55]	< 0.001	1.71 [1.21–2.42]	0.003
*General health factors*				
Access to health services when needed^b^	0.60 [0.44–0.83]	0.002	0.63 [0.44–0.91]	0.013
Detectable viral load^b^	1.33 [1.00–1.77]	0.050		
*Interpersonal factors*				
Sexual violence^b^	0.86 [0.50–1.50]	0.600		
Physical violence^b^	1.28 [1.02–1.61]	0.033	1.24 [0.96–1.60]	0.094

^a^A highly marginalized Vancouver community with high rates of poverty, unstable housing, substance use, survival sex work, and HIV infection

^b^In the last 6 months

^c^In the last 4 weeks, and restricted to 2019 February

Bivariate and multivariable logistic regression analyses with GEE were also performed to investigate the association between pain and the quality-of-life outcomes (Table 3). For each quality-of-life outcome, a confounder model approach was used in which all variables included in the full multivariable explanatory model for pain were considered confounders. As a first step in our confounder model fitting process, we assessed the relationship between all potential confounders described above and each outcome. Variables that were significantly associated with the outcome at a p < 0.10 level were included as potential confounders in the next step of model fitting. Next, for each outcome, the most parsimonious model was determined using the process described by Maldonado and Greenland [43], in which potential confounders were removed in a stepwise manner, and variables that altered all of the associations of interest by < 5% were systematically removed from the model. The final set of confounders included in the adjusted models are provided in footnotes in Table 3. The adjusted models used a complete case approach to remove observations with any missing data to ensure the model selection process was performed with nested models using constant sample size.

**Table 3:** Bivariate and multivariable odds ratios for the association between major or persistent pain in the last 6 months and outcome measures among cohort of 335 women living with HIV in Metro Vancouver, Canada

	Good self-rated health^ac^		Health interfered with social activities^bd^		Health interfered with general function^bd^	
	OR [95%CIs]	AOR [95%CIs]	OR [95%CIs]	AOR [95%CIs]	OR [95%CIs]	AOR [95%CIs]
Major or persistent pain^a^	0.56 [0.44–0.72]**	0.64 [0.48–0.84]*	2.51 [1.95–3.23]**	2.21 [1.63–2.99]**	3.63 [2.87–4.60]**	3.24 [2.54–4.13]**

All multivariable models were adjusted for the following variables: age, sexual minority identity, depression, suicidal ideation, non-injection opioid use, access to health services, physical violence, and food and/or housing insecurity

^a^In the last 6 months

^b^In the last 4 weeks

^c^Confounders in final model: depression (0.61 [0.47–0.79], p < 0.0001), suicidal ideation (0.65 [0.51–0.84], p < 0.01)

^d^No confounders in final model

^*^p < 0.01

^**^p < 0.001

Data are presented as unadjusted odds ratios (ORs) or adjusted odds ratios (aORs) with 95% confidence intervals (CIs). All p values are two-sided.

## Results

### Sample characteristics

Overall, 335 WLWH in SHAWNA were included in our sample, who contributed 1632 observations over 5 years from September 2014 to August 2019. The median number of follow-up visits in our study sample is five (interquartile range: 2, 7) with 2.4% of the sample having 10 visits. At baseline, 48.1% (161/335) of participants reported pain in the last 6 months, of which 19.1% (64) reported undiagnosed pain and 26.9% (90) reported that they had managed pain with criminalized drugs. Of those who reported pain, 64.0% (103/161) reported good self-rated health, 38.2% (58) reported interference of health with social activities, and 82.2% (125) reported interference of health with general function. Across all study visits, 77.3% (259) of participants reported pain at least once in the last 6 months, with 46.3% (155) experiencing any undiagnosed pain and 53.1% (178) managing pain with criminalized drugs.

Table 1 summarizes the characteristics of women in our sample at their baseline interview, stratified by major or persistent pain in the last 6 months. The median age of participants was 45 years (IQR 38–52 years). Capturing fluidity in sexual and gender identity over time, 40.6% (136) reported sexual minority and 10.5% (35) reported gender minority identity at any study visit, with 6.6% (22) identify as trans women (including transgender women, transsexual women, and other trans feminine identities) and 2.7% (9) reporting non-binary identity. Indigenous women comprised 55.5% (186) of the sample and were overrepresented compared to the population of British Columbia (5.9% in 2016 by Statistics Canada). Among Indigenous women, 14.5% (27/186) were Two-Spirit. Overall, 10.2% (34) were otherwise racialized women and 34.3% (115) were white women.

### Correlates of pain

ORs and aORs for bivariate and multivariable logistic regression using GEEs to assess the relationships between explanatory variables (excluding discrimination and HIV stigma measures) and pain in the last 6 months are shown in Table 2. Multivariable logistic regression analysis using GEEs indicated that age (aOR 1.04 [1.03–1.06] per year increase), food and housing insecurity (aOR 1.54[1.08–2.19] versus food and housing secure), depression diagnosis (aOR 1.34[1.03–1.75]), suicidal ideation (aOR 1.71[1.21–2.42]), and non-daily, non-injection opioid use (aOR 1.53[1.07–2.17] versus no non-injection opioid use) were associated with higher odds of pain, while daily non-injection opioid use (aOR 0.46[0.22–0.96] versus no non-injection opioid use) and increased access to health services (aOR 0.63[0.44–0.91]) were associated with lower odds of pain. In bivariate analysis, there was no significant association between detectable viral load, cannabis use, injection opioid use, unintentional overdose, sexual violence, or physical violence and major or persistent pain at p < 0.05, although viral load (p < 0.10), physical violence (p < 0.10), and less than daily cannabis use (p < 0.20) trended towards higher odds of pain.

### Association between pain and quality-of-life outcomes

Table 3 presents ORs and aORs for bivariate and multivariable logistic regression with GEE models for the association between pain and quality-of-life outcomes. Pain was associated with lower odds of excellent, very good, or good self-rated health versus fair or poor self-rated health (aOR 0.64[0.48–0.84]), and with increased odds of participants reporting that their health interfered with social activities (aOR 2.21[1.63–2.99]) or general function (aOR 3.24[2.54–4.13]).

## Discussion

Three-quarters of WLWH in our setting reported pain at ≥ 1 study visit, with half of WLWH reporting undiagnosed pain or pain self-managed with criminalized drugs. Correlates of pain included food and housing insecurity, depression, suicidal ideation, non-daily non-injection opioid use, and difficulty accessing health services. Pain was associated with reduced self-rated health, social participation, and general level of function. These outcomes are consistent with findings that chronic pain increases psychological distress and decreases self-efficacy, resulting in the avoidance of physical, occupational, and social activities [37]. They add to growing evidence that pain plays a crucial role in health-related quality of life among WLWH [58, 68].

The high proportion of participants managing pain with criminalized drugs in our study is concerning as there is an drug toxicity crisis in British Columbia characterized by contamination of the criminalized drug supply. Unintentional overdose now represents the major driver of mortality in PLWH in the province [66]. While we did not observe an association between pain and overdose, our data are limited to before 2019, after which the annual rate of drug toxicity deaths in British Columbia increased from 19.4 to 42.7 per 100,000 in 2022 [8]. Additional investigation is required to determine whether WLWH and pain are currently at risk for overdose in the context of an increasingly contaminated and criminalized drug supply.

High-risk opioid use is both a facilitator of pain (e.g. through opioid-induced hyperalgesia or increased tolerance to prescription analgesics) and an outcome (e.g. when opioids are used for symptom management) [50, 74]. Chronic pain and opioid use stigma also interact to restrict healthcare access (e.g. when individuals requesting pain treatment are dismissed as “drug-seeking”), and are compounded by colonial violence against Indigenous peoples, racism, and marginalization associated with im/migrant status, sexual orientation, and/or gender identity [76]. While the use of criminalized drugs for pain management in our cohort is consistent with an association between non-daily, non-injection opioid use and increased odds of pain, daily non-injection opioid use was unexpectedly associated with reduced odds of pain while no association was observed between injection opioid use and pain. Further work is needed to clarify these relationships. Daily non-injection opioid use may be effective for pain management in this population, which would be consistent with weak evidence that long-term prescription opioid use can provide clinically significant relief for chronic non-cancer pain [56]. In addition, daily opioid access may require lower levels of disability, allowing for greater access to care. It is also possible that WLWH using non-prescription opioids for pain management prefer to use non-injection routes of administration due to the shorter half-life of intravenous opioids.

While less than daily cannabis use trended towards higher odds of pain, a statistically significant association was not observed. Previous work demonstrates that many PLWH in Metro Vancouver may use cannabis for analgesia [14] and that cannabis is associated with reduced opioid use in people who use drugs (PWUD) with chronic pain [36]. However, these study cohorts consisted exclusively of PWUD who reported higher rates of cannabis use than our cohort and may have been more reliant on non-prescription drug use for pain management.

The associations between depression and suicidal ideation with pain are consistent with evidence that pain severity in PLWH is correlated with depressive symptoms [73]. Like substance use, depression has a bidirectional relationship with pain: depression may result in dysfunctional cognitive appraisals of pain and activate a sensitized stress response that facilitates chronic pain development, while pain itself is a negative affective state that increases the risk for depression [41]. Indeed, a qualitative study of PLWH and pain suggests that emotional and physical distress may be experienced indistinguishably [50]. While we conceptualized depression as a correlate of pain, future research could explore the potential role of depression in the other associations explored in this study, for example, as a mediator or moderator between pain and quality of life.

Structural conditions had a major impact on shaping experiences of pain in WLWH in our study. Half our cohort reported food and housing insecurity, which was associated with increased odds of pain compared to those who were food and housing secure. This is consistent with findings that half the patients at a Vancouver community-based chronic pain clinic lived below the poverty line [44]. Chronic pain can precipitate disability, limiting employment and socioeconomic status [44], while poverty can conversely increase the risk of developing chronic pain through allostatic overload [41] and may intersect with other facilitators of chronic pain. The associations between pain and poverty, substance use, and depression—as well as the documented interrelationships between these factors [16]—brings into question whether they may be conceptualized as a syndemic among WLWH. A syndemic describes the intersection of social, structural, and health issues that reinforce each other synergistically to increase disease burden, such as the “SAVA syndemic” of Substance Abuse, Violence, and HIV/AIDS among urban-dwelling women in the USA [52]. To identify high-impact interventional strategies, further work is needed to determine the extent to which poverty, substance use, depression, and chronic pain in WLWH may be mutually or serially causal and/or have interactive effects on functional outcomes.

Our results have important implications. The frequent use of criminalized drugs for pain management indicates that many WLWH may have difficulty accessing pain care. A previous examination of barriers to primary care in our study context concluded that equity-oriented approaches may improve access for WLWH [19]. The EQUIP framework, which operationalizes 4 dimensions of equity-oriented care (i.e. inequity-responsive care, trauma- and violence-informed care, culturally competent care, and contextually tailored care) [10], has been integrated into several HIV and primary care clinics in British Columbia [9, 33], although more work is required to upscale these services. The use of criminalized drugs for analgesia also highlights the importance of harm reduction in mitigating the risks of opioid use for WLWH. Based on our findings, we echo calls for expanded “safe supply” services to provide pharmaceutical-grade alternatives to toxic street drugs along with decriminalization to facilitate destigmatization of substance use and remove police-related barriers to healthcare access [24, 28, 45, 65].

The association between depression and pain in WLWH highlights the importance of dually indicated interventions, including psychotherapy. Cognitive behavioural therapy is a first-line treatment for depression associated with improved pain in PLWH [17, 71]). As conventional psychotherapy is predicated on Western colonial models of mental health [5] and two-thirds of our cohort were Indigenous or otherwise racialized, the promotion of Indigenous healing practices (e.g. access to Elders, traditional teachings, and land-based activities [63, 64]) and/or culturally adapted psychotherapeutic approaches may also be helpful for WLWH and pain. Unfortunately, low-barrier psychotherapy services are sparse in Metro Vancouver and more public investment is required to improve access. As mental distress is a common response to systemic inequities like poverty, racism, and colonial violence [55], these services must be situated within a wider framework of structural reform.

The importance of structural interventions is emphasized by the relationship between food and housing insecurity and pain in WLWH. Previous work has established that the most persistent barrier to managing chronic illness occurs when individuals do not have their basic needs met [6]. Income assistance and basic income have both been found to improve food and housing security [1, 23, 32], which may empower WLWH to better manage chronic pain. Housing-specific interventions may take the form of rental assistance, tenant advocacy services, and supportive housing environments that are safe, stable, and affordable. To meet the needs of cis and trans WLWH, it is imperative that supportive housing be low-barrier, family-oriented, integrated with other health and social services, and rooted in principles of trauma-informed care, harm reduction, and gender-responsiveness [79].

Our study has several limitations. First, participants indicated whether they experienced “major or persistent pain” in the last 6 months, a metric that includes severe acute pain, likely overestimates the prevalence of chronic pain among WLWH, and does not indicate changes in pain over time. It is conversely possible that the 6-month recall period may underestimate the occurrence of chronic pain due to recall bias, although this is less likely as a previous meta-analysis found no significant difference in the prevalence of pain reported by PLWH over 3-month to 6-month recall periods [59]. Ultimately, the prevalence of pain in our cohort is within the range reported for chronic pain by previous ART-era studies of PLWH and WLWH [59]. Second, stigmatized conditions (e.g. suicidal ideation) may have been under-reported by participants. However, questionnaires were designed with community consultation and administered by trained peer interviewers to optimize participant safety, allowing us to observe a high prevalence of other stigmatized conditions (e.g. criminalized drug use). Third, our relatively small sample size may have prevented us from identifying all associations with pain, but using repeated measures among participants over time effectively increased our statistical power. Fourth, as self-reported pain was assessed over the last 6 months while quality-of-life outcome measures were assessed in the last 6 months (self-rated health) and in the last 4 weeks (health interference in social activities and general function), it is therefore possible that the explanatory variable and outcomes could have overlapping time periods or that pain could have occurred 5–6 months before negative quality of life was assessed. Moreover, causality in the direction that we posit cannot conclusively be established. However, we feel that major or persistent pain is likely to have had an impact on quality of life within the 6-month period, particularly as there is extensive qualitative and quantitative evidence suggesting a directional association between pain and quality of life [15, 26, 46]. Finally, our results may not be generalizable to all WLWH in or beyond Metro Vancouver. However, we feel that our community-based outreach strategy allowed us to engage diverse participants, including those not previously connected to HIV care and whom we subsequently referred for services.

## Conclusion

In conclusion, a high proportion of WLWH experienced pain correlated with depression, suicidality, opioid use, food and housing insecurity, and poor access to health services. Pain had significant consequences for self-rated health and quality of life. The high proportion of WLWH in our study who reported the use of criminalized drugs for analgesia underscores the importance of harm reduction including access to a safe regulated supply and decriminalization in response to the opioid epidemic. Our study results also emphasize the need for structural change enabling WLWH and pain to meet their basic needs, including those related to food and housing security. While further work will elucidate the interrelationships between pain, substance use, and depression, our findings suggest that equity-informed pain services and anti-poverty interventions are urgently needed to improve quality-of-life outcomes in WLWH.

## Data Availability

In accordance with data access policies, our ethical obligation to research that is of the highest ethical and confidentiality standards, and the highly criminalized and stigmatized nature of this population, anonymized data may be made available on request to researchers subject to the UBC/ Providence Health Ethical Review Board, and consistent with our funding body guidelines (NIH, CIHR). The UBC/ Providence Health Ethics Review Board may be contacted at 604-683-2344.
